# Tadalafil protects from doxorubicin-induced heart failure by attenuating PKG Iα disulfide dimerisation

**DOI:** 10.1186/2050-6511-16-S1-A75

**Published:** 2015-09-02

**Authors:** Oleksandra Prysyazhna, Joseph R Burgoyne, Philip Eaton

**Affiliations:** 1 King's College London, London, SE1 7EH, UK

## Background

Phosphodiesterase (PDE) 5 inhibitors, which prevent cGMP degradation and therefore elevate its level in the heart, have been shown to protect the myocardium in several animal models of heart failure and in human trials. cGMP binding to protein kinase G (PKG) results in its classical activation and also prevents disulfide dimerisation-dependent activation of this kinase. Doxorubicin (DOX) is an anti-cancer drug which commonly causes cardiomyopathy, the pathogenesis of which is associated with increased oxidant production and apoptosis. We hypothesised that oxidative activation of PKG Iα may mediate DOX-induced cardiotoxicity, and PDE5 inhibitors may be protective because they elevate cGMP which then limits the oxidative activation.

## Methods

To test the hypothesis that PKG Iα disulfide dimerisation is involved in the development of DOX-induced cardiomyopathy, we used PKG Iα knock-in (KI) mice that express C42S kinase that cannot be oxidant-activated. DOX (15mg/kg) or saline was injected intraperitoneally to wild type (WT) and KI littermate male mice and hearts were analysed 5 days later. In separate experiments mice were co-treated with DOX and tadalafil (1mg/kg 30 min before DOX and then daily). Hearts were assessed by echocardiography, and then tissues were histologically assessed for apoptosis by quantitative TUNEL staining. Proteins involved in the pro-survival signalling and apoptosis were also analysed by immunoblotting analyses of these tissues.

## Results

Doxorubicin treatment caused loss of myocardial tissue and depression of left ventricular function, reduced pro-survival signaling and increased apoptosis in WT mice. Thus in the WT exposed to DOX ejection fraction decreased by 17%, apoptotic nuclei increased 7-fold, the BCL2/Bax ratio decreased, and the phosphorylation status of each of ERK, AKT and GSK3β significantly decreased. In contrast, KI mice were markedly resistant to this dysfunction. In WT mice, co-administration of tadalafil with doxorubicin reduced PKG Iα oxidation caused by doxorubicin, and also protected against cardiac injury and loss of function. KI mice were again resistant to doxorubicin cardiotoxicity, and therefore tadalafil did not cause any additional protection. Doxorubicin treatment resulted in a decrease of RhoA (Ser188) phosphorylation in WT, but not KI mice, stimulating its GTPase activity to activate ROCK. These pro-apoptotic events were attenuated in WTs co-administered with tadalafil.

## Conclusion

These data suggest that PKG Iα disulfide formation transduces oxidant signals that arise in the heart during DOX chemotherapy into a pro-apoptotic signal, leading to cardiomyopathy. The PDE5 inhibitor tadalafil abrogates this maladaptive signaling through cGMP elevation, which binds the kinase to limit its oxidation. Consistent with this, mice lacking the C42 redox-sensor in PKG Iα are therefore protected from heart cell apoptosis and injury otherwise induced by DOX.

